# Preoperative Cerebellar Mutism Syndrome in an Adult Female With a Fourth Ventricle Epidermoid Cyst: A Case Report and Review of the Literature

**DOI:** 10.7759/cureus.105731

**Published:** 2026-03-23

**Authors:** Alvaro Guerra-Krebs, Aldo F Berti

**Affiliations:** 1 Neurological Surgery, Neuronics Perú, Lima, PER; 2 Neurological Surgery, Instituto de Gamma Knife del Pacífico, Lima, PER

**Keywords:** cerebellar mutism, compression of fourth ventricle, giant epidermoid cyst, neuro radiology, posterior fossa surgery

## Abstract

Cerebellar mutism syndrome (CMS) is typically a postoperative complication of posterior fossa tumor resection, especially in pediatric medulloblastoma. We report a rare case of preoperative CMS in a 67-year-old woman with a fourth ventricle epidermoid cyst. She presented with dizziness, gait instability, emotional lability, and speech arrest. MRI revealed a multilobulated mass occupying the fourth ventricle and extending into the cerebellar hemispheres. After total surgical resection and ventriculoperitoneal shunt placement, histopathology confirmed an epidermoid cyst. The patient progressively regained speech and gait stability, with complete speech recovery and no recurrence at three-year follow-up. This case suggests that mass effect on the dentato-thalamo-cortical pathway can produce reversible preoperative CMS in adults and that recognition of this entity is essential for surgical planning and prognosis.

## Introduction

Epidermoid cysts are benign cystic lesions that account for only 0.1-1.8% of intracranial lesions [1,2]. These rare intracranial masses arise from gastrulation dysembryogenesis with disruption of neural tube closure between the third and fifth week of gestation [3]. Their most common location is the cerebellopontine angle (CPA), where two-thirds of these masses are found, followed by the fourth ventricle [2]. The most typical symptom is insidious headaches in a middle-aged patient, although symptoms will depend on their localization and mass effect. CPA lesions tend to manifest with cranial nerve involvement, whereas fourth ventricle lesions manifest more commonly with cerebellar symptoms [2,4]. As the cerebellum and its efferent pathways are in close contact with these fourth ventricle lesions within the posterior fossa, their surgical resection can place these important neurological structures at risk of injury [2].

Cerebellar mutism syndrome (CMS) has been described as a complication of the resection of posterior fossa tumors and consists of a constellation of signs and symptoms due to cerebellar damage, mainly alteration of linguistic production and emotional liability, although manifestations of cerebellar and brainstem dysfunction may also be present [5]. This condition has been widely described as a complication of surgical resection, especially in children with medulloblastoma [6]. Nonetheless, its preoperative presentation, especially in adults, has not been described.

We present the case of a 67-year-old woman with an infrequent epidermoid cyst located in the fourth ventricle who presented preoperative CMS, emphasizing its pathophysiologic implications and favorable prognosis after decompression. To our knowledge, this may represent the first reported case of preoperative CMS in an adult with a fourth ventricle epidermoid cyst.

## Case presentation

Preoperative findings

A right-handed 67-year-old woman with no past medical or surgical history attended our outpatient clinic in January 2022, referred by a neurologist for presenting a 10-month history of vomiting, dizziness, and loss of balance. Due to these symptoms, a brain MRI was performed in November 2021, showing a posterior fossa lesion occupying the fourth ventricle in its entirety, compatible with an epidermoid cyst. Her only current medication was pregabalin, prescribed by her neurologist, and paracetamol as needed for headaches.

Upon physical examination, the patient appeared depressed and tearful, showing emotional liability. She had to use a wheelchair due to severe dizziness. When spontaneous gait was evaluated with assistance, she was found to have cerebellar ataxia. Deep tendon reflexes were diminished in all four extremities. She demonstrated speech arrest alternating with markedly reduced, low-volume, slurred, and hesitant speech. She was diagnosed with a giant posterior fossa epidermoid cyst for which she was prescribed dexamethasone 2 mg three times a day (TID) to reduce the edema and mass effect, and a new contrasted brain MRI was indicated.

**Figure 1:**
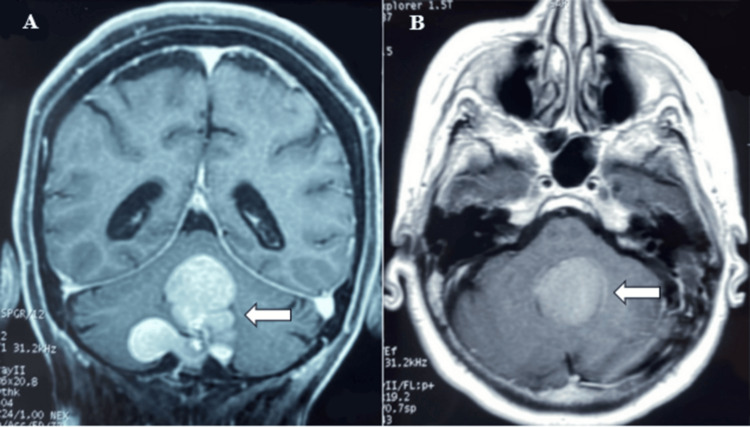
Preoperative contrast-enhanced T1-weighted MRI in coronal (A) and axial (B) views (arrow). The images demonstrate a 4-cm-wide T1-hyperintense multilobulated mass occupying the entire fourth ventricle, with extension through the foramen of Magendie, into the Luschka recesses, and into the bilateral cerebellar hemispheres, more prominently on the right. Moderate ventricular dilatation is also present.

The new brain MRI showed a slight increase in lesion size with signs of ventricular dilatation (Figure 1). The dizziness and gait instability mildly improved with the corticosteroid therapy, but speech arrest symptoms persisted. A posterior fossa craniectomy for lesion resection was scheduled, and informed consent was obtained with a clear understanding of the surgical technique, probable complications, and outcomes.

Surgical intervention

A suboccipital craniectomy for lesion removal was performed at the Navy Medical Center, Lima, Peru, achieving complete resection of the posterior fossa lesion located in the fourth ventricle, right cerebellar hemisphere, with extension into the foramen of Magendie and cisterna magna, causing extensive dilatation of the fourth ventricle. There was no compression of the brainstem or superior cerebellar peduncles, only adhesion to the left inferior cerebellar peduncle. Three days later, a ventriculoperitoneal shunt was placed as a consequence of postoperative hydrocephalus. She tolerated both procedures well.

**Figure 2:**
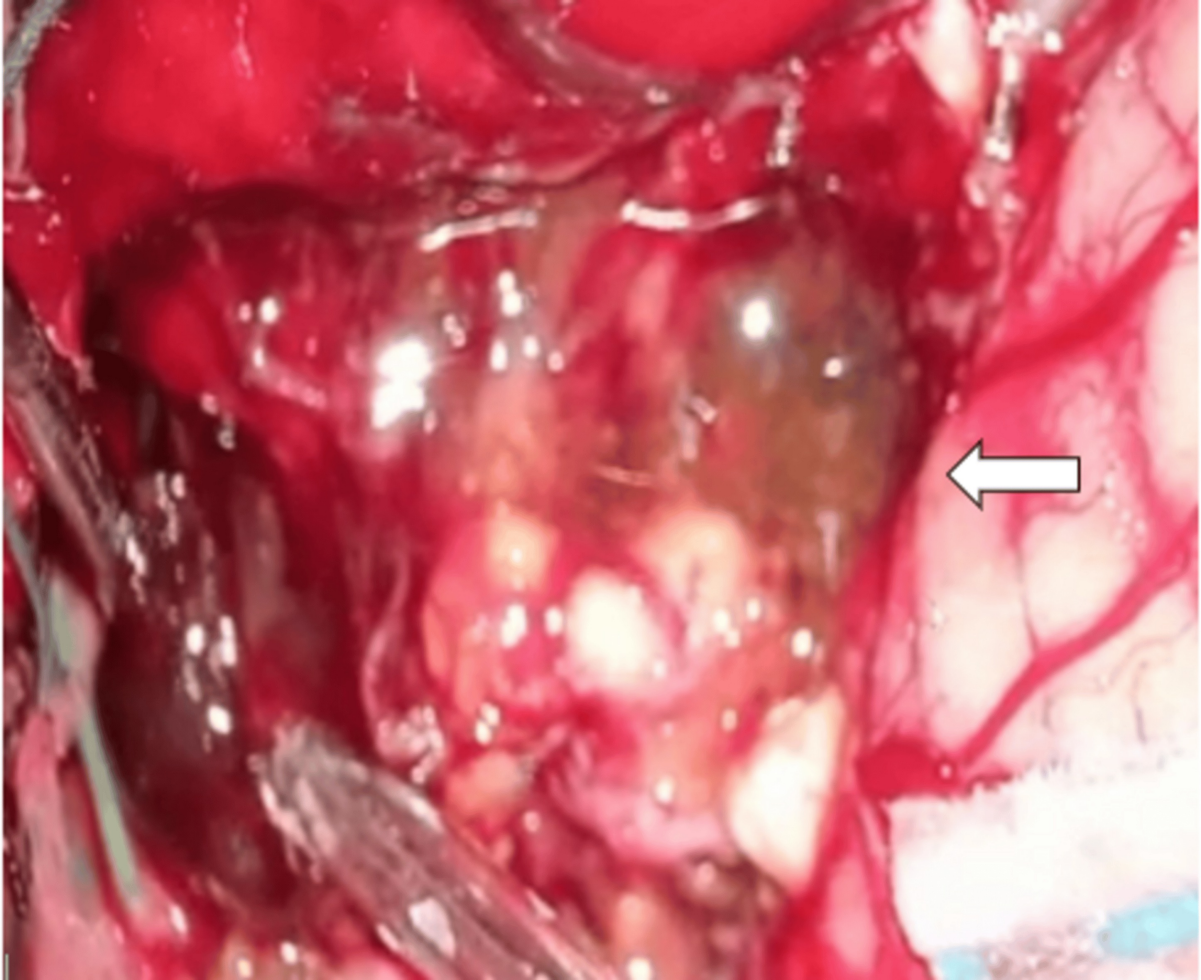
Intraoperative appearance of the multilobulated fourth ventricle epidermoid cyst (arrow).

Macroscopically, the resected lesion was described as a tough, irregularly shaped, brown-grayish mass of tissue (Figure 2). Histopathological analysis of the lesion showed cystic structures with stratified epithelioid cell lining and focal presence of a granular layer. These findings were compatible with an epidermoid cyst. A mild inflammatory infiltrate accompanied by a xanthogranulomatous reaction was also reported.

Postoperative course and follow-up

At the three-month follow-up appointment in April 2022, the patient had significant improvement in the cerebellar symptomatology and was able to walk with assistance. There was minimal right dysmetria. Extraocular movements were normal without nystagmus, and hearing and facial movements were also preserved. The patient initially showed improvement in speech initiation and had become more communicative and answered questions clearly. She appeared to be anxious, so evaluation by a psychiatrist was recommended. Postoperative brain MRI showed total lesion resection with postoperative changes at the surgical bed and adequate ventricular dimensions.

At a later follow-up appointment in August 2022, seven months after the surgery, the patient was very active and speaking spontaneously with adequate language fluency. Although gait instability had improved since her last visit, it was still present, for which she continued to use a cane for assistance when standing up and walking. Mild sporadic headaches were also reported. After evaluation by a psychiatrist, she was prescribed mirtazapine and pregabalin. A new brain MRI again showed total lesion resection without signs of focal recurrence and adequate ventriculoperitoneal shunt positioning without signs of hydrocephalus.

**Figure 3:**
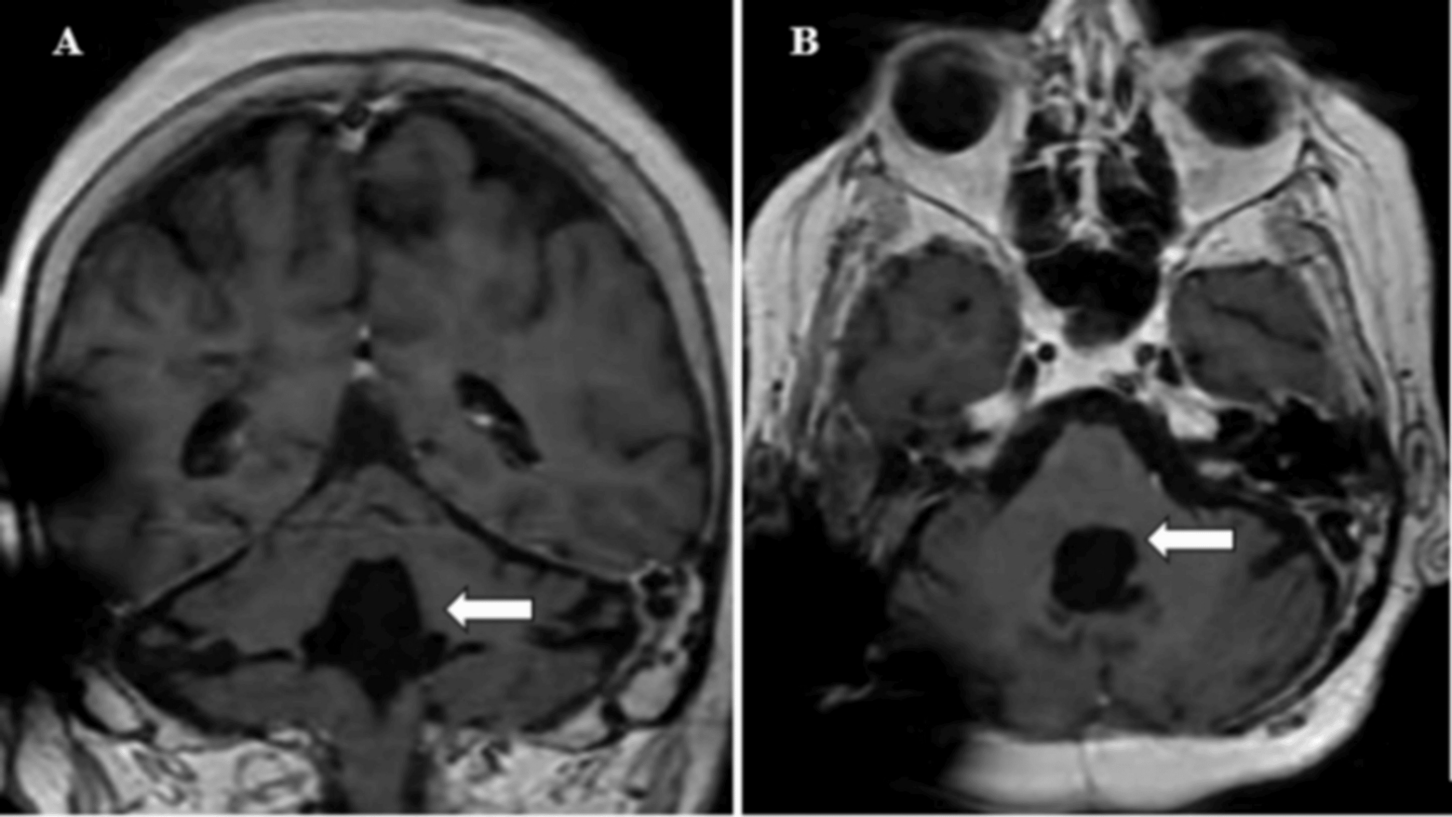
Three-year postoperative follow-up contrast-enhanced T1-weighted MRI in coronal (A) and axial (B) views (arrow). The images demonstrate postoperative changes with no evidence of lesion recurrence in the posterior fossa. A compensatory dilation of the fourth ventricle is present. The lateral and third ventricles are normal in size, with no hydrocephalus.

As of August 2025, more than three years after surgery, gait stability has significantly improved, and the latest MRI shows a decompressed fourth ventricle with absence of lesion recurrence and adequate ventriculoperitoneal shunt placement (Figure 3). The patient’s progressive symptom improvement and imaging findings were suggestive of a favorable prognosis.

## Discussion

Epidermoid cysts are congenital intracranial lesions externally covered by a thin capsule of keratinized stratified squamous epithelium that desquamates, breaking down into cholesterol and keratin, which allows it to grow very slowly [7,8]. Their growth is related to invasion of cisternal spaces and attachment to neurovascular contents, which correlates to the presentation of symptoms commonly in middle-aged or older patients, reflecting its location [2,4]. These lesions are observed on MRI with low-intensity signal on T1-weighted images, high-intensity signal on T2-weighted images, and FLAIR heterogeneous signal with moderate peripheral enhancement and diffuse hypersignal [8]. When radiological findings deviate from these characteristics, the lesion is referred to as a “white epidermoid cyst.” These lesions appear hyperintense on T1-weighted images, as observed in our patient´s preoperative images (Figure 1), likely due to high protein content [9]. Although malignant transformation is uncommon, it should be considered when there is a rapid progression of symptoms or contrast enhancement within the cyst is seen on imaging [10]. Surgery is the most recommended treatment, consisting of simple aspiration and total or subtotal resection. Nonetheless, recurrence rates vary from 8 to 25%, and malignant transformation within five years is described to occur more frequently in cases of subtotal excision [8,11,12].

As mentioned earlier, a potential complication of resecting posterior fossa tumors, such as epidermoid cysts, is CMS. The invasion of the fourth ventricle, such as in this case, has been described as one of the main risk factors for this condition, along with tumor size, vermis incision, and brainstem and superior cerebellar peduncle invasion [13]. Although it is described as a postoperative condition, there is a record of a pediatric case reported where CMS manifested preoperatively in a three-year-old with a posterior fossa juvenile pilocytic astrocytoma, demonstrating that this phenomenon can occur even before surgical intervention [14]. However, to our knowledge, no such cases of preoperative CMS have been reported in adults.

The pathophysiology of CMS is believed to involve disruption of the proximal efferent cerebellar pathway, which includes the dentate nucleus, the superior cerebellar peduncle, and its decussation in the mesencephalic tegmentum. These fibers travel towards the red nucleus and the thalamus, forming the dentato-thalamo-cortical pathways that connect the cerebellum to frontal and parietal cortical regions. One proposed mechanism is cerebral-cerebellar diaschisis, in which loss of excitatory impulses from the cerebellum to areas of the cerebral motor, premotor, and prefrontal cortex regions leads to both motor and cognitive function impairment. Other mechanisms thought to disrupt these pathways include edema, perfusion deficits, and cerebellar vermis injury [15,16]. In our patient, the large epidermoid cyst occupying the fourth ventricle and extending into adjacent cerebellar hemispheres likely caused direct compression of these critical structures, explaining the preoperative manifestation of mutism, emotional lability, and gait instability. The progressive improvement of these symptoms following decompression supports the reversibility of this dysfunction when mass effect is relieved. We acknowledge that distinguishing CMS from severe cerebellar dysarthria can be challenging, particularly in cases with markedly reduced speech output; however, the presence of speech initiation deficits and associated behavioral changes in our patient supports the diagnosis of CMS.

Fourth ventricular epidermoid cysts commonly present with cerebellar dysfunction and, less frequently, abducens nerve involvement, as described by Kumar et al. in their case series and systematic review, as well as with headache, nausea, and vomiting, as reported by Ahuja et al. [7,17]. As shown in Table 1, most reported cases present with cerebellar symptoms such as ataxia and headache. In contrast, our patient exhibited additional behavioral and language alterations consistent with CMS, distinguishing this case from those reported. Furthermore, the T1 hyperintensity observed in our case differentiates it radiologically from typical epidermoid cysts and is consistent with a “white epidermoid cyst,” as previously described.

**Table 1:** Reported cases of fourth ventricular epidermoid cysts, including the present case.

Author (Year)	N	Age/Sex	Clinical Presentation	Imaging Features	Treatment	Extent of Resection	Outcome
Kumar et al. (2021) [7]	1	36/F	Headache, vertigo, ataxia	T1 hypointense / T2 hyperintense	Midline suboccipital craniotomy	Complete	Stable
Kumar et al. (2021) [7]	1	46/F	Diplopia, ataxia, headache	T1 hypointense / T2 hyperintense	Midline suboccipital craniotomy	Partial	Improved
Kumar et al. (2021) [7]	1	26/F	Headache, nystagmus	T1 hypointense / T2 hyperintense	Midline suboccipital craniotomy	Complete	Stable
Bensamma et al. (2022) [18]	1	52/M	Ataxia, dysmetria	T1 hypointense / T2 hyperintense	Midline suboccipital craniectomy	Complete	Stable
Bensamma et al. (2022) [18]	1	49/F	Headache, ataxia	T1 hypointense / T2 hyperintense	Midline suboccipital craniectomy	Partial	Improved
Costea et al. (2025) [19]	1	57/F	Ataxia, diplopia, nystagmus, dysarthria	T1 hypointense / T2 hyperintense	Midline suboccipital craniectomy	Partial	Improved
Present case	1	64/F	Headache, ataxia, emotional lability, speech alterations	T1 hyperintense / T2 hyperintense (“white epidermoid”)	Midline suboccipital craniectomy	Complete	Improved

It is described in pediatric patients that recovery from mutism in postoperative CMS occurs in almost all cases, although language and communication deficits tend to improve without fully resolving [15]. In contrast, our patient demonstrated complete recovery of language fluency, suggesting that the underlying mechanism was non-destructive, likely secondary to reversible mass effect rather than injury to cerebellar efferent pathways, which may have contributed to the favorable prognosis, as similarly reported by Chen et al. [14]. Prognosis is also influenced by surgical factors. In this context, surgical approaches that minimize injury to midline cerebellar structures are critical, as the telovelar approach has been associated with a lower risk of postoperative cerebellar mutism and truncal ataxia compared to the transvermian approach [19,20].

## Conclusions

Epidermoid cysts are rare fourth ventricle lesions whose surgical resection can result in CMS. This condition, although widely described as a postoperative consequence of posterior fossa tumor resection, can also present preoperatively when specific pathways are compromised due to mass effect. As this is a single case, broader series are needed to characterize the prognosis of adult preoperative CMS more fully. It is important for neurosurgeons to identify it before surgical intervention and understand its possible mechanism, as recognizing preoperative CMS may aid in counseling patients and predicting recovery after decompression.
